# Can Twitter be used to predict county excessive alcohol consumption rates?

**DOI:** 10.1371/journal.pone.0194290

**Published:** 2018-04-04

**Authors:** Brenda Curtis, Salvatore Giorgi, Anneke E. K. Buffone, Lyle H. Ungar, Robert D. Ashford, Jessie Hemmons, Dan Summers, Casey Hamilton, H. Andrew Schwartz

**Affiliations:** 1 Center on Continuum of Care in Addictions, Perelman School of Medicine, University of Pennsylvania, Philadelphia, Pennsylvania, United States of America; 2 Positive Psychology Center, Department of Psychology, University of Pennsylvania, Philadelphia, Pennsylvania, United States of America; 3 Computer and Information Science Department, University of Pennsylvania, Philadelphia, Pennsylvania, United States of America; 4 Center on Continuum of Care in Addictions, University of Pennsylvania, Philadelphia, Pennsylvania, United States of America; 5 Department of Emergency Medicine, Perelman School of Medicine, University of Pennsylvania, Philadelphia, Pennsylvania, United States of America; 6 Department of Computer Science, Stony Brook University, New York, New York, United States of America; Tampere University of Technology, FINLAND

## Abstract

**Objectives:**

The current study analyzes a large set of Twitter data from 1,384 US counties to determine whether excessive alcohol consumption rates can be predicted by the words being posted from each county.

**Methods:**

Data from over 138 million county-level tweets were analyzed using predictive modeling, differential language analysis, and mediating language analysis.

**Results:**

Twitter language data captures cross-sectional patterns of excessive alcohol consumption beyond that of sociodemographic factors (e.g. age, gender, race, income, education), and can be used to accurately predict rates of excessive alcohol consumption. Additionally, mediation analysis found that Twitter topics (e.g. ‘ready gettin leave’) can explain much of the variance associated between socioeconomics and excessive alcohol consumption.

**Conclusions:**

Twitter data can be used to predict public health concerns such as excessive drinking. Using mediation analysis in conjunction with predictive modeling allows for a high portion of the variance associated with socioeconomic status to be explained.

## Introduction

Excessive alcohol consumption, the third-leading preventable cause of death, is an important risk factor for many chronic diseases and accounts for an estimated 87,798 deaths yearly in the United States [1,2]. The 2014 National Survey on Drug Use and Health (NSDUH) reports that 60.9 million participants surveyed were binge drinkers (drinking five or more drinks on the same occasion on at least one day in the past 30 days) and 16.3 million reported heavy alcohol use (binge drinking on 5 or more of the past 30 days) [3]. Excessive (binge and heavy) alcohol consumption is associated with serious social and health consequences (e.g., automobile accidents, unintended injuries, cognitive problems, and suicide) [4]. The question of how best to measure alcohol consumption patterns and excessive alcohol consumption dates back to at least the 1920’s [5]. The three most prominent strategies include 1) asking participants to list their recent alcohol consumption occasions; 2) asking participants to summarize their alcohol consumption patterns; and 3) using alcohol sale and shipment data [6,7]. One challenge in measuring excessive alcohol consumption is the difficulty in gathering survey data—which traditionally has relied on national phone surveys. Surveys are costly, time-consuming, and susceptible to participant recall bias, socially desirable responding, and the researcher unintentionally biasing participant responses [8].

More flexible and significantly less expensive alternatives for population-level assessment utilize big data analysis of online media language [9,10]. Online data sources provide a relatively new resource for monitoring and understanding public health problems, including heavy and excessive alcohol consumption. There are many advantages of using social media data over traditional public health assessment tools. Social media data are real-time and can be collected and analyzed quickly. Researchers can observe discussions between individuals in their natural environment without the presence of the researcher. Additionally, individual statements are made as they occur—thereby reducing the memory recall biases that are common with national cross-sectional surveys. Google search queries have been used to measure trends in chronic disease risk, cancer screening, influenza incidence, new diagnoses of HIV infection and interest in synthetic cannabinoids and cathinones [9,11–15]. Due to the magnitude of content produced online, researchers can also use sophisticated natural language analysis methods to mine user-generated social media content to better understand health outcomes and health behaviors. For example, Twitter data have been used to track influenza symptoms, nicotine consumption, estimate alcohol sales volume, and measure depression, life satisfaction, HIV prevalence, and heart disease mortality at the county level [10, 16–26]. The large magnitude of user-generated social media content available allows researchers to use sophisticated natural language analysis methods to better understand health behaviors and outcomes. Natural language analysis has been used to detect poor healthcare quality; adverse drug events, and mental health status [27–30]. Paul and Dredze [20] analyzed Twitter user messages to measure different population public health characteristics. They applied the Ailment Topic Aspect Model to these tweets and were able to automatically extract a variety of public health data from tweets. Specifically, they were able to track illnesses over time (syndromic surveillance), measure behavioral risk factors, examine illnesses by geographic region, and analyze symptoms and medication usage. While these studies have demonstrated the power of Twitter to examine many health behaviors at the county level, the platform has yet to be used for predicting county level substance use and misuse patterns.

The purpose of this study was to leverage the power of Twitter communications to better understand excessive alcohol consumption at the county level. More specifically, we asked: 1) Are linguistic patterns on Twitter predictive of county-level excessive alcohol consumption? 2) How does Twitter content compare with and mediate demographic and socio-economic variables for predicting county-level excessive alcohol consumption? and 3) What linguistic patterns are most highly associated (both positively and negatively) with excessive alcohol consumption at the county level?

Twitter is a free social media platform that allows millions of users to send and receive each other’s “tweets” (i.e., short messages limited to 140 characters). We selected Twitter because of its national appeal and easy availability. As of the end of 2015, Twitter had over 65 million active monthly U.S. users and processed over 500 million “tweets” per day [31,32]. Since a significant portion of Twitter data is location-tagged, we can examine county-level variations of Twitter language with excessive alcohol consumption.

## Methods

The study was reviewed and approved by the University of Pennsylvania IRB-8. The review board found the study to be exempt due to not pertaining to human subjects research. Additionally, it should be noted that the authors do not have permission from Twitter to redistribute the tweets used in this study, though all tweets are publicly available via the Twitter platform.

### Data sources

We used data from the 1,384 U.S. counties and county equivalents (henceforth “counties”) for which excess alcohol consumption rates, county-level socioeconomic and demographic variables, and at least 40,000 tweeted words were available.

#### Twitter data

A random 1% of Twitter data was collected between October 2011 and December 2013, totaling 2.24 billion tweets using the TwitterMySQL Python package. This package directly connects to the official Twitter Streaming API (application programming interface) and allows users to download random Tweets, and associated information such as date, retweet information, user profiles, etc., in real time. TwitterMySQL was developed internally and has since been made open source (available at github.com/dlatk). Using the geolocation methods described in Schwartz et al. [33], we used self-reported location information in user profiles (from approximately 20% of tweets) and latitude/longitude coordinates (available for approximately 2% of tweets) to map tweets to U.S. counties. This resulted in 138 million county-mapped tweets across 3,146 counties. Limiting the analysis to counties for which at least 40,000 words were available, a more stringent limit than that recommended by Schwartz et al. [34] to insure an adequate sample of a county’s language, reduced this number to 1,667 counties.

Several steps were taken to extract features (i.e. independent variables) from the county tweet data. First, we split tweets (i.e. sequences of letters) into words using a publicly available tokenizer^1^ which was designed to capture typical language as well as other social media content such as emoticons (e.g. “:)”, “<3”) and hashtags (e.g. “#weed”) as “words”. Word frequencies were then summed at the county-level and used to find the relative frequencies of groups of related words, known as “topics.” Specifically, we used a social media-based set of 2000 topics derived from the MyPersonality Facebook data set (approximately 15 million posts) [33]. The topics were automatically discovered using Latent Dirichlet Allocation (LDA), a probabilistic technique [33]. This technique uses a generative model which assumes that each document (in this case, Facebook message) contains a distribution of topics, which is in turn assumed to be a distribution of words. The end result of LDA is a set of topics, with weights for each word belonging to the topics, denoted as p(topic|word). Words that are weighted highly within the same topic are those which have appeared in similar contexts. For example, the words *research*, *paper*, and *final* were all weighted highly within one particular topic. The particular set of 2000 topics which we use here were derived previously over social media [33] using the Mallet software package which estimates the latent variable of the topic using Gibbs sampling [35,36]. When running Mallet, all hyperparameters were set to their default value except *alpha*, a prior on the expected topics per document, *was set to 3*. This was chosen under the assumption that Facebook messages contain fewer topics than standard documents, such as blog posts or newspaper articles, where LDA was originally applied. These topics have previously been applied successfully in capturing county life satisfaction and heart disease mortality from tweets [10, 21].

The probability of topic usage per county, *P(topic|document)*, was derived using:

$$P(topic|document)=\sum_{tok\in topic}P(topic|tok)\times P(tok|document)$$ (1)

where *document* represents a county in this case. Here *P(tok|document)* was estimated from the relative frequency of tokens (words) in the county (as described above) and *P(topic|tok)*, the probability of a topic given the token, was derived though the LDA process. Thus, for each of the 1,667 counties, scores for the 2000 pre-specified topics formed the independent variables in our statistical analysis. The topic scores for each county as well as the topics themselves have been open-sourced and are available at at www.wwbp.org/data.html.

#### Excess alcohol consumption data

**Fig 1:**
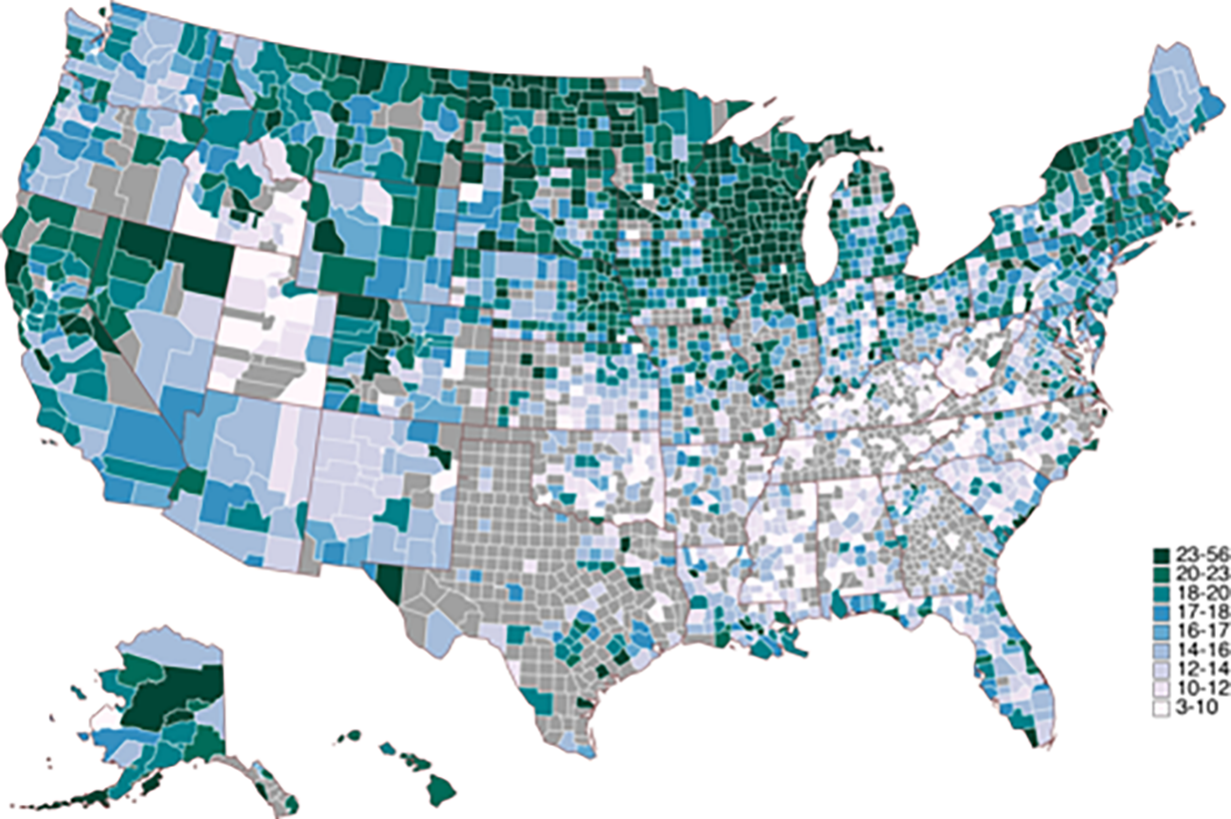
Excess alcohol consumption by county.

The Behavioral Risk Factor Surveillance System (BRFSS) is a population-based cross-sectional telephone and cell phone health survey of U.S. adults, aged ≥18 years, conducted by state and territorial health departments in conjunction with the Centers for Disease Control and Prevention. From the BRFSS (2006–2012), we obtained the county-level prevalence of self-reported binge drinking and heavy drinking [37] Excessive alcohol consumption was defined as having drunk more than two drinks per day on average (for men) or more than one drink per day on average (for women) or having drunk 5 or more drinks during a single occasion (for men) or 4 or more drinks during a single occasion (for women). Further limiting our set of counties to those with excessive alcohol consumption scores reduced our county set to 1,431. Excessive alcohol consumption by county is presented in Fig 1.

#### Demographic and socioeconomic data

**Fig 2:**
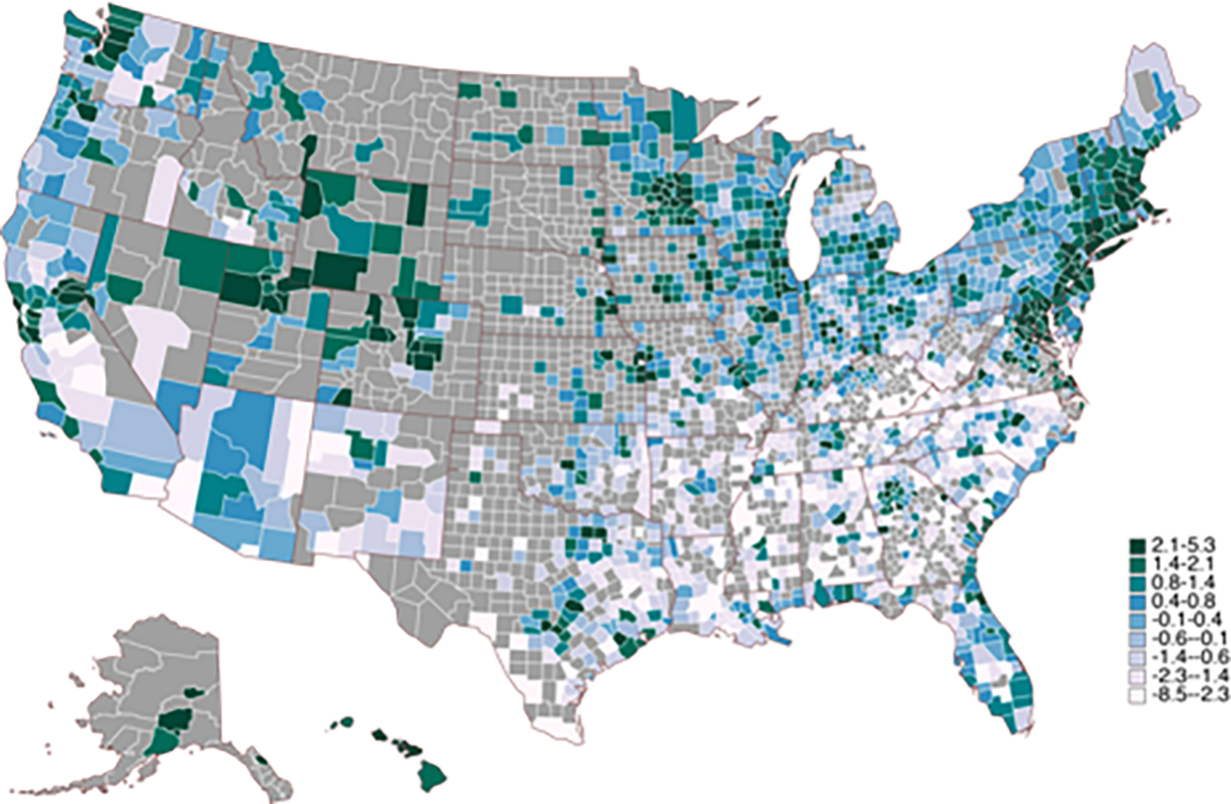
Socioeconomic index by county.

We obtained percentages of female, foreign born, African-American and Hispanic as well as age percentages (19 bins) from the U.S. Census Bureau [38,39]. County-level measures of log income, percentage of married residents, high school and college graduation rates, and unemployment were obtained from the American Community Survey [40]. Because income and education are highly correlated, we created a composite county-level *socioeconomic index* (Fig 2) by averaging standardized log income and standardized high school graduation rates. Such averaging often results in a stronger single predictor [41]. Limiting our analysis to counties with income and education rates left 1,384 counties. The socioeconomic index then forms our starting point for mediation analysis—using topics to understand the relationship between county socioeconomics and excessive alcohol consumption.

### Statistical methods

To explore the relationship between language and excessive alcohol consumption, we performed 3 statistical analyses: (1) *Predictive modeling*, to quantify how well Twitter language predicts excessive alcohol consumption, (2) *Differential Language Analysis (DLA)* to identify which features on Twitter correlate with excessive alcohol consumption, and (3) *Mediating Language Analysis* to reveal how language use mediates the relationship between socioeconomics and excess alcohol consumption. The entire analytical pipeline (from feature extraction to modeling, DLA and mediation) used the Differential Language Analysis ToolKit (DLATK) Python package which is available at github.com/dlatk [42]. While demographic and socioeconomic variables may predict alcohol consumption, they do not explain *why* this is the case. That is, we know that being white is associated with drinking more alcohol than being black, but it is presumably something correlated with race that is the causal driver rather than race itself. The Twitter topics that mediate the relation between demographics and alcohol consumption provide suggestions of that ‘lurking’ causal mechanism—of the many word topics that correlate with demographics, only some also predict alcohol consumption.

#### Predictive modeling

Predictive models of county excessive alcohol consumption, with and without socio-demographic controls, were created using well-established regularized regression techniques from the field of machine learning [43]. Specifically, we used a pipeline of feature selection, principal components analysis, and ridge regression [10]. During feature selection we first removed all low variance features and then features that were not correlated with excess drinking at a family-wise error rate alpha of 60. This preprocessing pipeline of feature selection and principal components analysis was used to avoid overfitting since our model included more independent variables (2000 topic frequencies) than units of analysis (1,384 counties). Therefore, we did not apply this pipeline to any model which did include the topic features, i.e., models with only socio-demographic variables.

Across three sets of models, we varied the features *X* (i.e. independent variables) and considered excessive alcohol consumption as the dependent variable *y*. For all models using linguistic features (2000 Facebook topics), we used the standard ridge regression equation:

$$\beta =(X^{T}X+\lambda I)X^{T}y$$ (2)

where the regularization term *λ* was set to 1,000 and chosen via cross validation. For models using only non-linguistic features (demographic and socioeconomic variables), we used the standard ordinary least squares (OLS) linear regression equation:

y=Xβ+ϵ (3)

For *model 1*, we used only the 2000 Twitter topics with a ridge regression (Eq 2). For *model 2*, we used (a) demographics alone (percentage of female, African American, Hispanic, foreign born and married residents as well as four age bins: 1–14, 15–29, 30–60 and 60+), (b) the socioeconomic index alone, all socioeconomic variables (log income, high school and college graduation rates, and unemployment), and (c) all non-language variables combined, all via OLS linear regression (Eq 3). Finally, for *model 3*, we combined the demographic and socioeconomic variables with the Twitter language. In order to distinguish the small number of socio-demographic variables from the 2000 language features we first regressed the socio-demographic variables against excess drinking, via a OLS linear regression (Eq 3), and used the residuals for building the models with the language features via ridge regression (Eq 2).

We measured prediction performance using the Pearson correlation between the model predictions of excessive alcohol consumption and BRFSS reported rates. We used 10-fold cross validation [43], in which counties were partitioned into 10 parts, or “folds”. A predictive model was built by fitting the independent variables to the dependent variables over 9 of the 10 folds, and then evaluated by determining how well the model predicted the outcomes for the remaining 10th fold. This procedure was repeated 10 times and the results were averaged together to determine the overall prediction performance. Significance of differences between models’ performance was compared using a paired t-test over the models’ error.

#### Differential language analysis

We used Differential Language Analysis (DLA) [20] to identify language that characterizes excessive alcohol consumption. In DLA the topic variables were individually correlated with excessive alcohol consumption and the socioeconomic index using an ordinary least squares regression. The variables were first standardized and we fit linear regression, using OLS linear regression (Eq 2), for each topic with and without the inclusion of the socioeconomic index as a covariate, to excessive alcohol consumption. The coefficient associated with the topic variable is reported. Because we made multiple comparisons (2000 topics per outcome), we applied a Benjamini–Hochberg correction to the significance threshold [44]. We note that while less stringent than Bonferroni correction, Benjamini–Hochberg is justified under thoroughly rigorous statistical principles. Additionally, if nothing is significant under Bonferroni then nothing will be significant under Benjamini–Hochberg either.

In order to visualize the resulting topic correlations, we include word clouds for the top (highest correlation coefficient) significant results. These word clouds display the top 15 most prevalent words within a topic sized according to their posterior likelihood.

#### Mediating language analysis

We examined the relations between the socioeconomic index and excessive alcohol consumption as mediated by the Twitter topics. Here, we make no assumptions as to which of the 2000 topics mediates the relation. Instead, we take a rigorous big data approach and consider all 2000 Twitter topics as potential mediators, again correcting for multiple hypothesis tests using Benjamini-Hochberg.

For each topic in our topic set we considered the mediating relations among the socioeconomic index (as the independent variable), topic (as mediator), and excessive alcohol consumption (as the dependent variable). Each mediation follows the recommended standard three-step process [45]. First, we regressed socioeconomic index *x* with excessive alcohol consumption *y* (path *c*) and socioeconomic index with the topic *m* (path *a*) via:

$$y=cx+\beta_{1}+\epsilon_{1}$$ (4)

$$m=ax+\beta_{2}+\epsilon_{2}$$ (5)

Then in a single multi-variate model, we regressed both the topic (the mediator *m*; path *b*) and the socioeconomic index (the independent variable *x*; path *c’*) with excessive alcohol consumption:

$$y=c^{\prime }x+bm+\beta_{3}+\epsilon_{3}$$ (6)

to determine how much the topic mediates the relationship between SES and excessive alcohol consumption. The effect size of the mediation is assessed as the reduction between the direct relationship of SES with excessive alcohol consumption and the relationship once the topic is taken into account, *mediation size = c–c’*. We test for significance, using a Sobel *p* [46]. Because we repeated this analysis over a large number of mediators (2000 different topics), we applied the Benjamini-Hochberg procedure to correct the p values for false discoveries.

## Results

### Direct correlations

Table 1 shows correlations between excessive alcohol consumption and the standard demographic (female, foreign born, African-American, Hispanic, and age percentages) and socio-economic variables (log income, high school and college graduation rates, as well as log income, percentage of married residents, and unemployment). Excessive alcohol consumption was most positively correlated with having graduated high school, log income, and having graduated college, while it was most negatively correlated with percentage of African Americans, women, and unemployment. The final column contains all cross correlations for our composite socioeconomic index, which besides correlating with its member variables, had the strongest correlation with excessive alcohol consumption of any variable. The high correlation between excess drinking and these variables (*r = 0*.*05* to 0.*49)* suggest their use as a strong baseline of structural controls to attempt to out-predict with our Twitter language features.

**Table 1:** Cross correlations (Pearson *r*) Between Excess Alcohol Consumption, Demographic and Socioeconomic Variables.

	Percent Female	Percent African American	Percent Hispanic	Percent Foreign Born	Percent Married	Log Income	Unem-ployment	High School Grad	College Grad	Socio-economic Index
Excess Drinking	**-.23**	**-.25**	**.05**	**.12**	**.06**	**.43**	**-.17**	**.45**	**.30**	**.49**
Percent Female		.30	-.10	-.01	-.08	-.06	-.09	-.01	.12	.04
Percent African American			.10	-.01	-.59	-.28	.21	-.34	-.07	-.34
Percent Hispanic				.74	-.09	.09	.09	-.34	.06	-.13
Percent Foreign Born					-.16	.37	-.02	-.12	.41	.15
Percent Married						.41	-.14	.27	-.06	.38
Log Income							-.37	.61	.67	.91
Unem-ployment								-.42	-.43	-.44
High School Grad									.63	.89
College Grad										.72

*Note*: For ease of inspection, correlations are color formatted, ranging from strongly positive (dark blue) to strongly negative (dark red). Cells in white are not significant, all other correlations significant at a Benjamini–Hochberg corrected significance level of *p* < 5x10^-4^.

### Prediction models

To test the hypothesis that Twitter language adds predictive values beyond standard socioeconomic and demographic variables, we compare models built on (1) the language alone, (2) demographics, (3) socioeconomics, as well as combinations of each All models were fit over one portion of data and tested on a held-out portion using a 10-fold cross-validation procedure as discussed in the methods under predictive modeling. Accuracy of each model is reported in Table 2 as the Pearson Product-Moment Correlation Coefficient between the BRFSS excess drinking variable and its prediction based on the given model over heldout test data. According to a paired t-test on model error, accuracy was significantly greater for the combined model (socioeconomic and demographic variables plus Twitter) than the model built only on the socioeconomic and demographic variables alone (*r* = 0.54 to *r* = 0.69; *t* = -9.35, *p* = 1x10^-6^).

**Table 2:** County-level excessive alcohol consumption prediction accuracy.

Independent Variable	Pearson r	Mean Absolute Error
Twitter language alone	0.65	2.70
Demographics	0.39	3.40
Socioeconomic index	0.48	3.27
All socioeconomic variables	0.47	3.22
Demographics and socioeconomics	0.54	3.08
Twitter language, demographics and socioeconomics	0.69*	2.61

*Note*: Prediction performance (Pearson *r*) using 10-fold cross validation.

* significant decrease in error over demographics and socioeconomics from paired t-test (*t =* -9.35, *p <* 1x10^-6^*)*.

### Differential language analysis

**Fig 3:**
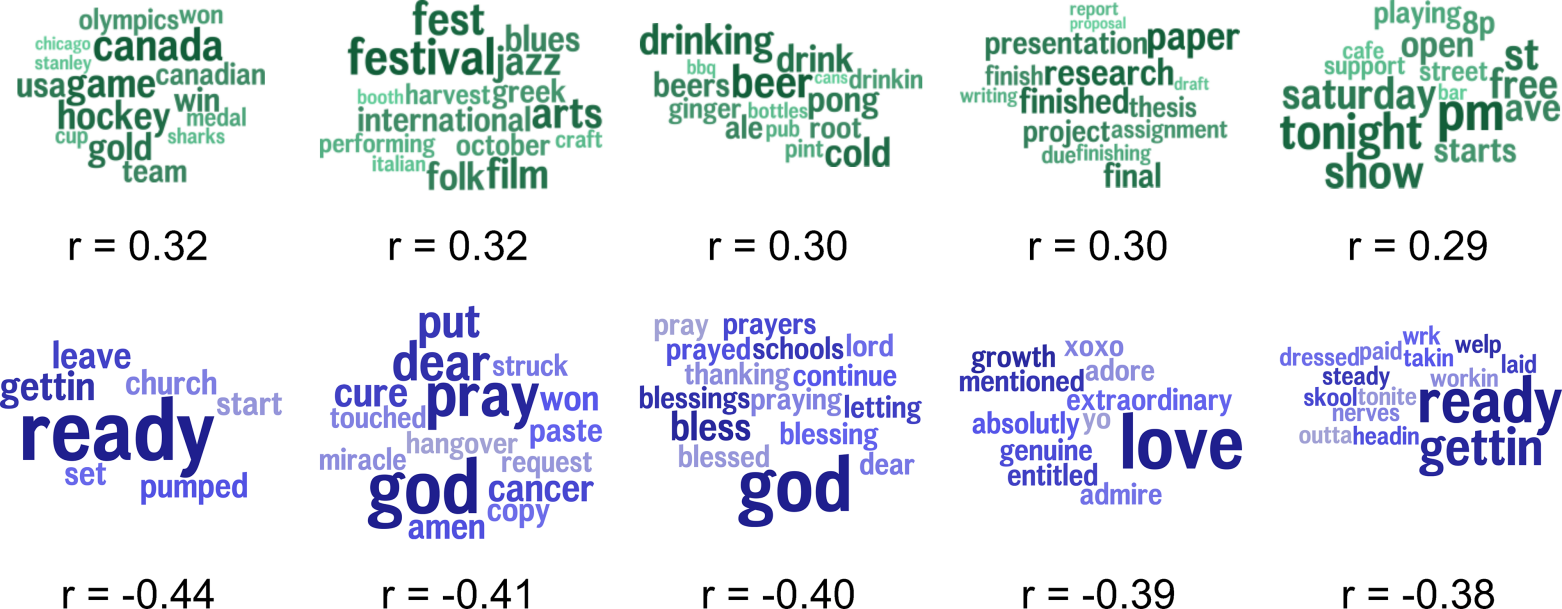
Topics correlated with excessive alcohol consumption. Topics in teal are positively correlated with excess alcohol consumption, while topics in blue are negatively correlated. The size of the word represents its relative frequency within the topic. Larger words are more frequent, while smaller words are less frequent. All correlations significant at a Benjamini–Hochberg corrected significance level of *p* < 1x10^-6^.

**Fig 4:**
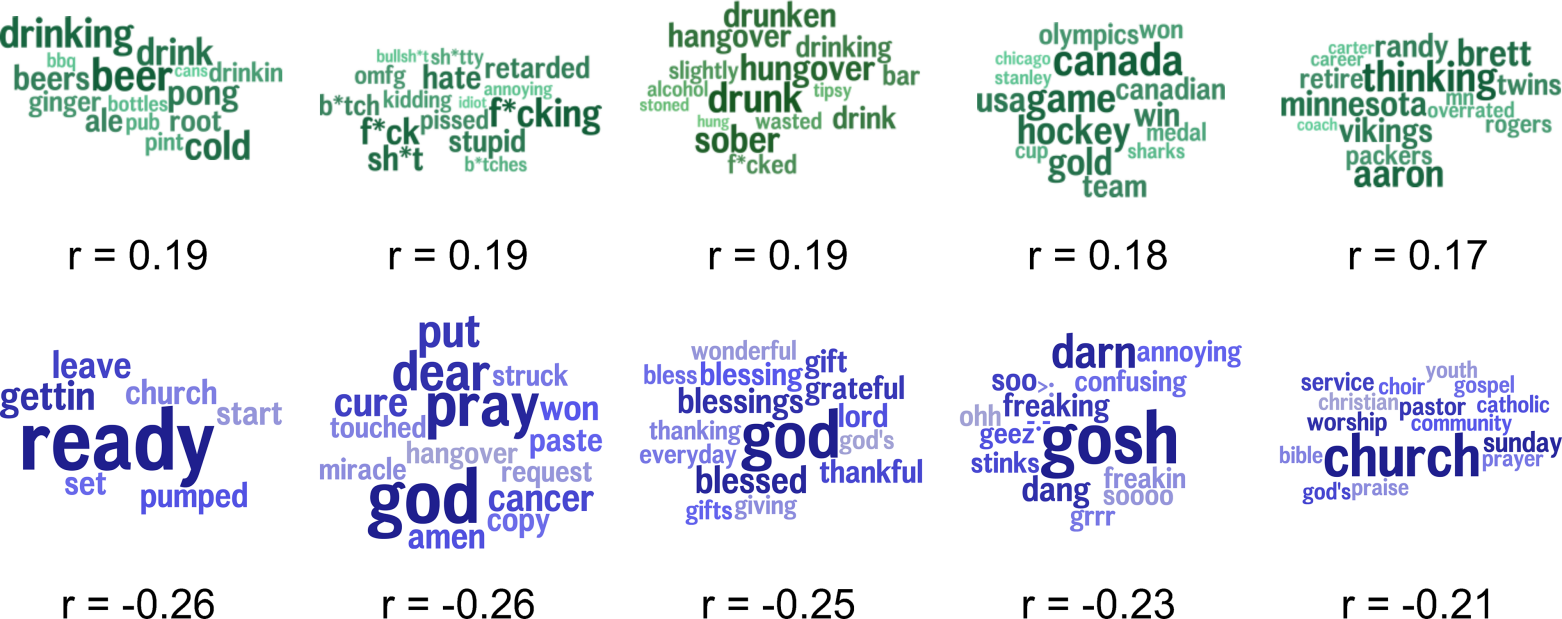
Topics correlated with excessive alcohol consumption controlled for the socioeconomic index. Topics in teal are positively correlated with the excess drinking, while topics in blue are negatively correlated. The socioeconomic index was used as a covariate in the regression. All correlations significant at a Benjamini–Hochberg corrected significance level of *p* < 1x10^-6^.

**Fig 5:**
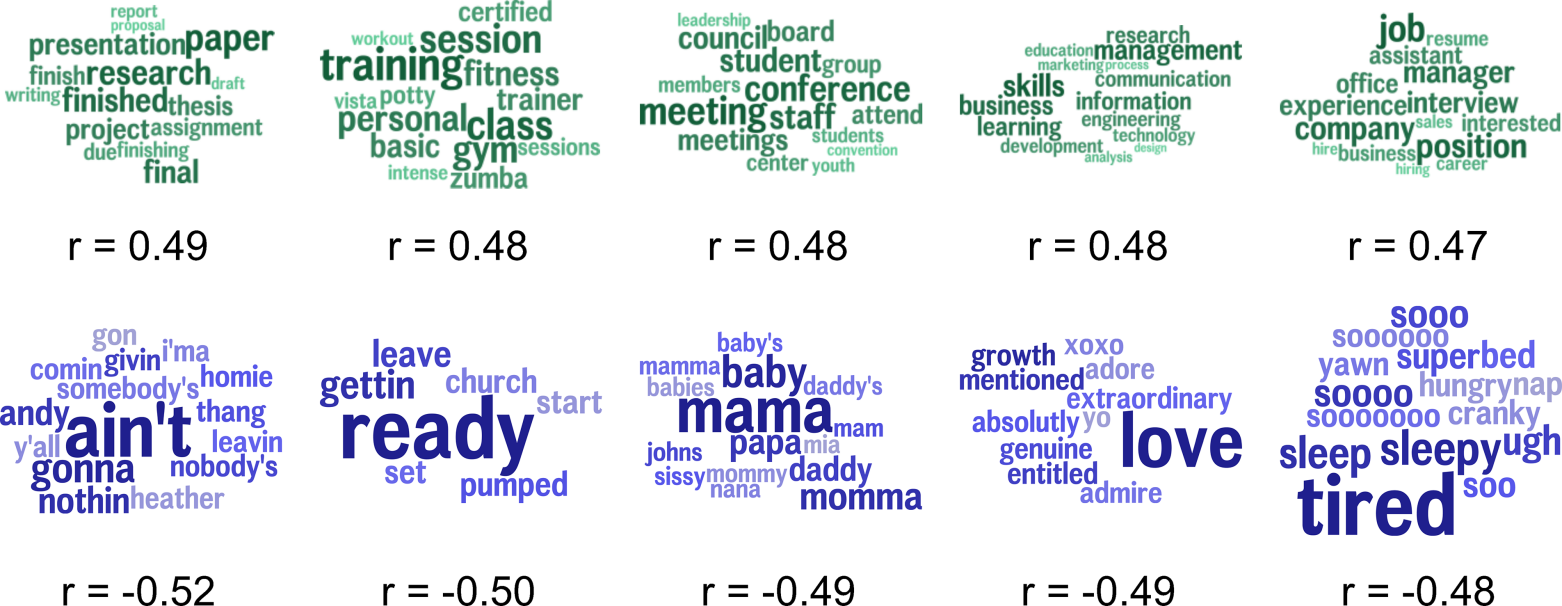
Topics correlated with the socioeconomic index. Topics in teal are positively correlated with the excess drinking, while topics in blue are negatively correlated. All correlations significant at a Benjamini–Hochberg corrected significance level of *p* < 1x10^-6^.

To assess specific linguistic features as markers of community excess drinking, we found those topics most highly correlated with excessive alcohol consumption as depicted in Fig 3. Since we considered 2,000 total topics, a Benjamini-Hochberg procedure was applied to correct significance tests for multiple hypotheses. Topics positively correlated with excessive alcohol consumption included *game*, *hockey*, *gold* (*r* = 0.32), *festival*, *jazz*, *film (r* = 0.32), *drinking*, *beer*, *drink (r* = 0.30), *research*, *paper*, *final (r* = 0.30) and *tonight*, *pm*, *saturday (r* = 0.29). Topics negatively correlated with excessive alcohol consumption included *ready*, *getting*, *leave (r* = -0.44), *god*, *dear*, *pray (r* = -0.41), *god*, *blessed*, *thanking (r* = -0.40), *love*, *adore*, *admire (r* = -0.39), and *ready*, *getting*, *tonight* (*r = -0*.*38)*. Topics most correlated with excessive alcohol consumption while controlling for the socioeconomic index are shown in Fig 4. Positively correlated topics include *beer*, *drink*, *drinking (r* = 0.19), *hate*, *stupid*, sh*t (r = 0.19), *drunk*, *hangover*, *drunken (r* = 0.19), *hockey*, *game*, *win (r* = 0.18) and *minnesota*, *thinking*, *brett (r* = 0.17). Negatively correlated topics include *ready*, *pumped*, *church (r* = -0.26), *god*, *pray*, *dear (r* = -0.26), *god*, *blessed*, *thankful (r* = -0.25), *gosh*, *dang*, *darn (r* = -0.23) and *church*, *community*, *sunday (r* = -0.21). Finally, Fig 5 shows topics most correlated with the socioeconomic index. Positively correlated topics include *paper*, *research*, *presentation* (*r* = 0.49), *training*, *class*, *gym* (*r* = 0.48), *meeting*, *conference*, *staff* (*r* = 0.48), *skills*, *management*, *business* (*r* = 0.48) and *company*, *interview*, *position* (*r* = 0.47). Negatively correlated topics include *ain’t*, *gonna*, *nothin* (*r* = -0.52), *ready*, *getting*, *leave* (*r* = -0.50), *mama*, *baby*, *momma* (*r* = -0.49), *love*, *growth*, *mentioned* (*r* = -0.49) and *tired*, *sleepy*, *sooo* (*r* = -0.48).

### Mediating language analysis

Mediating language analysis was used to better understand the relationship between county socioeconomics and excessive alcohol consumption by looking at the Twitter topics which best explain their relationship. Each topic was considered individually as a mediator, one at a time, of the relation between the socioeconomic index and excessive alcohol consumption: *c* is the correlation coefficient of the direct path between SES and drinking while *c’* represents the correlation strength once the topic was introduced as mediator.

**Fig 6:**
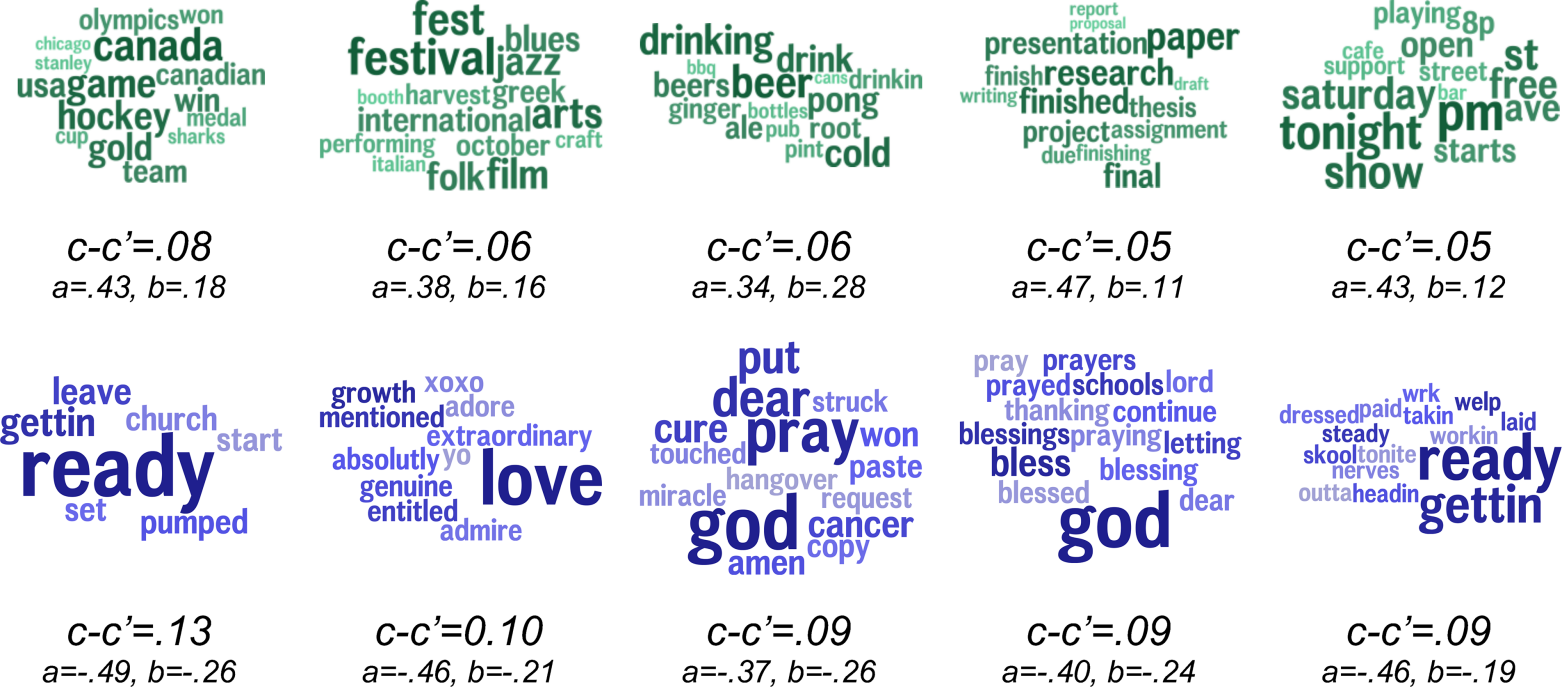
Top independent mediators. Topics in teal are positively correlated with the excess drinking, while topics in blue are negatively correlated. Topics are ordered from left to right by descending mediation effect size (*c-c’*). All mediation effects were significant at a Benjamini–Hochberg corrected Sobel significance level of *p* < 1x10^-4^.

The results of the mediating language analysis are shown in Fig 6. The topics are arranged in descending order of mediation effect size (*c—c’*) with topics positively correlated with excess alcohol consumption in the top row and negatively correlated topics in the bottom row. Topics with large mediation effect sizes are those which best explain the relationship between *SES* and *excessive alcohol consumption*. For example, the topic, *gettin*, *ready*, *leave*, not only correlates directly with less excessive alcohol consumption (r = -.44) and socioeconomic index (r = -.50) but also can be said to partially explain the variance between lower socioeconomics and lower excessive alcohol consumption since their relationship significantly decreases with the introduction of the topic (*c-c’ = 0*.*13; p* < 1x10^-6^). Similarly, increased discussion of *hockey*, *game*, *Canada* not only predicted more excessive alcohol consumption but also explained some of the variance between higher socioeconomics and more alcohol consumption (*c-c’ = 0*.*08; p* < 1x10^-5^). These mediations demonstrate that not only does language capture information above and beyond socioeconomics, but that language also directly captures part of the variance in socioeconomics that is related to excess drinking.

## Discussion

This paper sought to examine the relationship between language use on Twitter and county-level excessive alcohol consumption. Our results confirmed a number of correlates of excessive alcohol consumption known in the literature including positive correlation with income and high school and college graduation rates, and negative correlations with percentage African American, female and unemployed [47]. Most striking, perhaps, is the correlation of excessive county-level alcohol consumption with higher education, suggesting that higher educated counties drink more. This goes against stereotypes of the unemployed, depressed individual with an alcohol use disorder, but is consistent with recent research that reporting that the most educated Americans drink more alcohol [48]. Counties with higher unemployment also have lower rates of excessive alcohol consumption, further contradicting existing stereotypes. Counties with more female and more African-Americans drink less alcohol, possibly suggesting that the culture in these counties discourages problematic alcohol consumption. Future analysis should more closely examine these counties to see what cultures or practices might offer protective factors from excessive alcohol consumption and what interventions these suggest.

In terms of prediction, we found that the combined model (Twitter language plus sociodemographic variables) performed better than the model built using only the sociodemographic variables. Language expressed on Twitter revealed several community-level psychological characteristics that were significantly associated with excessive alcohol consumption. The topics most positively correlated with excessive alcohol consumption at the county level included sports, entertainment, educational activities, alcohol mentions, and specific times of the day and week. Negatively correlated topics related to religion, “getting ready” to engage in social activities (a major mediator of the effect of socio-economic status), and positive emotions such as love and admiration. This pattern also held true when we controlled for socioeconomic variables.

Topics positively correlated with excess alcohol consumption revolve in part around social activities where alcohol consumption is normative, such as sporting events, music, art, and food-related festivals. This lends itself to possible public health interventions. Festivals where alcohol is served may be a safe, socially accepted, place for binge drinkers to engage in excess alcohol consumption. Thus, these events may be particularly attractive to this group and encourage such individuals to relocate to such counties. However, easy access to such events where alcohol consumption is normative could also encourage binge drinking and increase rates of alcohol use disorders in a county. Social media interventions targeted at deterring excess alcohol consumption at such events may be particularly effective in reducing binge drinking and related problems. These interventions have the potential to be tailored to the specific event and have the potential to reach thousands of community members.

Counties with higher excessive alcohol consumption talked more about finishing assignments, presentations, and other types of academic and work-related projects. This may indicate that counties with a greater proportion of college students drink more. Non-linguistic analyses of social media usage habits of college students have previously had limited success in predicting drinking patterns [49], though alcohol-related social media marketing exposure has been found to positively predict college student alcohol consumption. Since most social media platforms, including Twitter, are popular among young adults who are also college students [35], the findings imply that interventions that target collegiate aged populations who use language predictive of excessive alcohol use may be effective. Additionally, social media intervention campaigns that model positive-language for college students may be potentially more effective than more traditional methods, serving as an additional protective factor in a digital environment.

Inhabitants of counties that score low on excessive alcohol consumption tweet more about religion than those in counties that drink more (i.e. religiosity appears to be a protective factor, consistent with prior work [50, 51] In addition, counties who have lower rates of excess alcohol consumption express planning and getting ready language, which could be indicative of a culture that is active and values self-control. Counties that do not consume alcohol excessively also have tweets that express gratitude and blessings, love and overall positive affect. These counties may have an infrastructure in place (e.g. attractive public spaces to meet and socialize), a high level of generalized trust such that members can fulfill belongingness needs and thus not feel a need to excessively drink to feel greater connection to others [52,53]

We asked whether linguistic patterns on Twitter predict county-level alcohol consumption and found that they do even above and beyond a large array of socio-economic and demographic variables. The major advantage of our language-based approach over traditional survey methods is that we can measure problem drinking unobtrusively and cheaply in large populations—more rapidly than traditional data sources. Social media-based language monitoring and insights may be especially useful in helping younger adults, who are particularly vulnerable to excess alcohol consumption and who also are heavy social media users. Longer term, we hope insights from social media language will drive formulations of interventions

Our results are, of course, not without limitations. They fundamentally correlational, and etiology is only suggested rather than shown. Further, Twitter itself is not a representative sample–younger individuals and those identifying as black or Latino are over-represented. Still, social media yields a quantifiable view into communities that is unobtrusive and natural, and we evaluated our model against an excessive alcohol consumption outcome based on representative samples. We have demonstrated that biased social media data can still be used to predict unbiased outcomes.

Traditional self-report questionnaires that measure excessive alcohol consumption are invasive and time consuming for responders, subject to reference biases, and limited to predetermined factors. Social media offers a large-scale, real-time, and unobtrusive window into health behaviors and attitudes. The words people use in their daily lives reveal important aspects of their social and psychological worlds. We found individuals on Twitter report on their alcohol use, their social interactions, and the events around them. These data streams occurred in real time and were marked with context, including location and can serve as an indicator of health status. In conclusion, we found this data source as a viable measure of county level excessive alcohol consumption.

This research takes an important step in advancing the science of measuring excessive alcohol consumption and has the potential to be used to improve public health. First, we provide a meaningful interpretation of county level excessive drinking using Twitter data. Second, we used this data source to provide new insights into the attitudes and interactions that promote excessive drinking (and protect from it). Third, this research will aid public health officials in identifying social media content, locations, and individuals who are at high risk for excessive alcohol consumption.

Future work should explore which factors in the culture (e.g. social cohesion, religion, future orientation, work ethic) of counties drive excessive alcohol consumption and which may be protective. Future work could also attempt to design and test interventions that reduce problem drinking such as targeting social norms for drinking at or to produce apps that alert social media users when their language suggests that they may be at risk of excessive alcohol consumption.
